# Viral etiologies in adult patients with encephalitis in Poland: A prospective single center study

**DOI:** 10.1371/journal.pone.0178481

**Published:** 2017-06-01

**Authors:** Marta Popiel, Karol Perlejewski, Agnieszka Bednarska, Tomasz Dzieciątkowski, Marcin Paciorek, Dariusz Lipowski, Monika Jabłonowska, Hanna Czeszko-Paprocka, Iwona Bukowska-Ośko, Kamila Caraballo Cortes, Agnieszka Pawełczyk, Maria Fic, Andrzej Horban, Marek Radkowski, Tomasz Laskus

**Affiliations:** 1 Department of Immunopathology of Infectious and Parasitic Diseases, Warsaw Medical University, Warsaw, Poland; 2 Department of Infectious Diseases, Warsaw Medical University, Warsaw, Poland; 3 Hospital for Infectious Diseases, Warsaw, Poland; 4 Department of Microbiology, Warsaw Medical University, Warsaw, Poland; Universitatsspital Basel, SWITZERLAND

## Abstract

Encephalitis is a severe neurological syndrome associated with high morbidity and mortality as well as long-term neurological sequelae. Despite being an important public health problem, very few extensive population-based studies were conducted so far in the world and none in Central Europe. Altogether 114 consecutive patients meeting the initial criteria for encephalitis were enrolled at the Warsaw Hospital for Infectious Diseases between June 2012 and July 2015. Eighteen patients were secondarily excluded from the analysis due to incomplete data or noinfectious cause. Potential pathogen sequences were searched for by molecular methods in the cerebrospinal fluid (CSF) and specific antibodies were detected in CSF and sera. An infectious agent was identified in 41 patients (42.7%). The most frequently diagnosed infections were Human herpesvirus 1 (HHV-1) (22 cases, 24%) followed by Enterovirus (6 cases, 6.3%), Varicella zoster virus (VZV) (5 cases, 5.2%), Tick-borne encephalitis virus (TBEV) (6 cases, 6.3%) and Cytomegalovirus (CMV) (2 cases, 2.1%). There were no cases of human adenovirus, Human herpesvirus 6 (HHV-6) or West Nile virus (WNV) infection identified. In 55 cases (57.3%) the cause of encephalitis remained unknown. Compared to patients in whom the diagnosis was determined the latter group contained more women, was less likely to manifest fever and had lower CSF pleocytosis (p < 0.05) In summary, we identified HHV-1 followed by Enterovirus, VZV and TBEV as the most common causes of encephalitis among adult patients in Poland. In a large proportion of patients the cause of encephalitis remained unknown.

## Introduction

Encephalitis is a severe neurological syndrome associated with high morbidity and mortality as well as long-term neurological sequelae and imposes therefore a severe burden on the health care systems [1]. In the US alone the estimated yearly costs of hospitalized encephalitis cases has been estimated at over half a billion dollars [2,3]. Encephalitis can be caused by a wide variety of infectious agents, most prominently viruses, although bacteria, fungi and protozoa were also implicated [1,4]. Clinically indistinguishable encephalitis cases could be also due to autoimmune factors and paraneoplastic conditions [5,6].

The most common causative agents of encephalitis are Human herpes virus type 1 (HHV-1); followed by varicella zoster virus (VZV) [1,3–5,7–9], while other herpesviruses like Epstein-Barr virus (EBV), cytomegalovirus (CMV) and human herpesvirus 6 (HHV-6) are less common and largely limited to immunocompromised patients [4,5]. Another important cause of encephalitis are arthropod-borne viruses (arboviruses) from the *Flaviviridae* family: West Nile virus (WNV) and Tick-borne encephalitis virus (TBEV) [10]. However, there are large differences between geographical regions with regard to the etiology of central nervous system (CNS) infections and the picture can change dynamically as new pathogens emerge and reemerge: for example West Nile virus (WNV) became prominent in the US but not in Europe, while Tick-borne encephalitis virus (TBEV) is a common cause of encephalitis in Europe, but not in the US [11,12]. Similarly, novel developments related to the increased travel and mobility were the spread of Japanese encephalitis virus (JEV) into India, Nepal and Australia and several outbreaks of Chikungunya virus infection in the Indian Ocean islands and India [13]. Neurovirulence could be also acquired by spontaneous mutations as was in the case of Enterovirus 71, which has gained the ability to invade the CNS causing meningitis and encephalitis [13,14]. However, nothwithstanding significant progress in diagnostics, in a large proportion of cases (40%-80%) the etiology remains unknown [5,15].

Despite being an important public health problem, very few extensive population-based studies have been conducted so far in the world and none in Central Europe. This scarcity of published data is partly due to the fact that the disease is rare and collecting of a meaningful number of cases is a long-term endeavor, while diagnostic procedures required are extensive and expensive.

We present the etiological and clinical results of a prospective single center population study encompassing 114 consecutive patients with encephalitis in Poland. To our knowledge this is the only large study from Central Europe and the largest single-center study on adult population.

## Patients and methods

Patients with encephalitis were prospectively enrolled at the Warsaw Hospital for Infectious Diseases between June 2012 and July 2015. This hospital is the single major center for neuroinfections serving central Poland including Warsaw (Mazowsze region).

Reporting of encephalitis cases is mandatory in Poland and in the years 2012–15 92% to 96% of all encephalitis cases reported each year in the Mazowsze Region were hospitalized in the above mentioned medical center.

The Mazowsze Region could be considered representative of the country: in 2015 the encephalitis incidence rate per 100,000 inhabitants was 0.49 while in 13 out of the the remaining 15 administrative regions it was between 0.22 and 0.71. The only exception were two heavily wooded regions in the Northeast, where the incidence was 1.87 and 7.31, respectively [16]. These latter regions are endemic for TBEV infection.

Encephalitis was defined as an acute onset illness with altered mental status or decreased level of consciousness or seizures or focal neurological signs together with at least one abnormality of the cerebrospinal fluid (CSF) (white blood cell count ≥ 4 cells/mm^2^ or protein level ≥ 40 mg/dL). Exclusion criteria were noninfectious CNS disease and meningitis without clinical manifestations of brain involvement. Patients below 18 years were not included in the study, since children with encephalitis are hospitalized in various other centers.

Written informed consent was obtained from all patients or from close relatives if the patient was unable to give consent due to his condition. However, this consent had to be confirmed once his condition improved. The study was approved by the Internal Review Board of the Warsaw Medical University.

The CSF and serum samples were collected from all patients at admission and whenever possible a follow up serum sample was collected after 4–6 weeks. All samples for the study were aliquotted and kept frozen at -80°C until analysis. Total RNA was extracted with TRIzol LS (ThermoFisher Scientific, Waltham, Massachusetts, USA) from 400 μl of whole CSF, while DNA was extracted using QIAamp DNA Mini Kit (Qiagen, Hilden, Germany). Extracted nucleic acids were concentrated when needed and suspended in 20 μl of water, 2 μl of which were subsequently used for each amplification reaction.

Human herpesvirus 1&2 (HHV-1 and HHV-2); Varicella zoster virus (VZV), Cytomegalovirus (CMV), Human herpesvirus 6 (HHV-6), Enteroviruses (Coxsackie A9, A16, B2, B3, B4, B5; ECHO 5, 6, 9, 11, 18, 30 and Entero 71), and Human adenovirus (HAdV) were detected in CSF samples using 'in house' real-time quantitative PCR (qPCR) or Reverse-Transcription real-time quantitative PCR (RT-qPCR) methods. For the detection of Tick-borne encephalitis virus (TBEV) and West Nile virus (WNV) qualitative Reverse Transcription PCR (RT-PCR) were used. The limit of detection (LOD) of each assay was determined by analysis of serial dilutions in the range of 10^1^−10^6^ viral copies or viral copy equivalents in the case of synthetic control templates [17–21]. Each dilution was prepared and analyzed in six independent replications. Probit analysis was used to calculate the LOD concentration [22]. Viral DNA or RNA copy numbers in clinical samples was quantified on the basis of threshold cycle (Ct) values of viral template calibrators. As a cut-off values, LODs of qPCR assays were used. The established LODs were as follows: for HHV-1 50 viral copies/reaction, HHV-2 70 copies/reaction, VZV 15 copies/reaction, Enteroviruses (for Coxsackie A9) 10 copies/reaction, HHV-6 30 copies/reaction, HAdV 20 copies/reaction, CMV 20 copies/reaction. RT-PCR for the detection of TBEV and WNV were capable of detecting 100 viral copies/reaction and 50 viral copies/reaction, respectively [23,24].

All the available CSF samples (both intial and follow up) were first tested by the above amplification assays and only when no pathogen could be detected serological analysis followed. Commercial serological tests were used to test CSF and paired sera samples (Serion ELISA classic HHV-1/2 IgG and IgM, Serion ELISA classic VZV IgG and IgM, Serion ELISA classic TBEV IgG and IgM, Institut Virion/Serion GmbH, Würzburg, Germany), anty-West Nile Virus IgM/IgG ELISA (Euroimmun, Lubeka, Germany). The tests were performed and interpreted following manufacturers' recommendations.

For statistical analysis continuous data were compared with Mann–Whitney *U* test, while proportions were analyzed by Fisher's exact test. Correlations were assessed by Spearman rank test. P ≤ 0.05 was considered to be statistically significant.

## Results

Altogether 114 patients meeting the initial criteria of encephalitis were enrolled between June 2012 and July 2015. Eighteen patients were secondarily excluded from the analysis: in nine clinical information was incomplete or the amount of biological material was insufficient for analysis, while in the remaining nine noninfectious causes were diagnosed. The remaining 96 patients were analyzed in the study. Follow up serum samples drawn after 4–6 wks were available in 53 (55%) of patients.

The etiologic investigation identified an infectious cause in 41 patients (42.7%). In 29 patients the pathogen was detected directly in the CSF by PCR or RT-PCR, while in 12 patients the diagnosis was based on either the presence of IgM antibodies in CSF (4 patients) or serum (2 patients) or a significant increase in serum antibodies over time (6 patients) (Table 1). The most frequently diagnosed infections were HHV-1 (22 cases, 24%) followed by Enterovirus (6 cases, 6.3%), TBEV (6 cases, 6.3%), VZV (5 cases, 5.2%), and CMV (2 cases, 2.1%). There were no cases of adenovirus, HHV-6 or WNV infection identified. In 55 cases (57.3%) the cause of encephalitis remained unknown. Only one patient was coinfected with two pathogens as both HHV-1 and Enterovirus sequences were detected in his CSF.

**Table 1:** Identified etiologic causes of encephalitis in Polish patients (n = 96).

Etiologic diagnosis	No. (%) of patients	Diagnosis based on molecular testing of CSF	Viral copies/mL (median; range)	Diagnosis based on serological testing		
				Specific IgM in CSF	Specific IgM in serum	≥ 4 fold antibody increase in paired sera over 4–6 weeks
Known etiology	41 (42,7)					
HHV-1	22 (24,0)^a^	19	3,450 (1,500–3,815,000)	-	-	3
VZV	5 (5.2)	2	1,250 and 750	-	-	3
CMV	2 (2.1)	2	1000 and 10,500	-	-	-
HHV-6	0 (0.0)	-		-	-	-
Enteroviruses^b^	6 (6.3)	6	875 (300–11,450)	-	-	-
TBEV	6 (6.3)	-		4	2	-
WNV	0 (0.0)	-		-	-	-
HAdV	0 (0.0)	-		-	-	-
Unknown etiology	55 (57.3)					

^a^One HHV-1 positive patient was coinfected with Enterovirus.

^b^The RT-PCR assay was capable of detecting Coxsackie A9, A16, B2, B3, B4, B5; ECHO 5, 6, 9, 11, 18, 30 and Entero 71.

HHV-1, human herpesvirus 1; HHV-6, human herpesvirus 6; TBEV, Tick-borne encephalitis virus; WNV, West Nile virus; HAdV, human adenovirus.

The major clinical features and the results of CSF analysis according to diagnosis are presented in Table 2. Among 96 patients only two were HIV-positive, while another 5 were receiving immunosuppressive drugs and 7 had cancer (Table 2). During the presenting illness the majority of patients had fever, headache and altered mental state, and a significant minority had decreased consciousness, focal neurological signs and seizures. Nineteen patients (20%) presented with stiff neck. The median hospital stay was 12 day (range 5–97). Only one death occurred—this was a TBEV-infected patient on pharmacological immunosuppression due to a liver transplant. While viral sequences were not detected in his CSF, they were found in autopsy brain tissue.

**Table 2:** Clinical and CSF data of 96 patients with encephalitis according to etiology.

Parameter^a^	HSV-1 (n = 22)	VZV (n = 5)	Enterovirus (n = 6)	TBEV (n = 6)	CMV (n = 2)	All identified (n = 41)	Unknown (n = 55)
Male	16 (73)^b^	2 (40)	4 (67)	6 (100)	2 (100)	30 (73)^b^	24 (44)
Age, median years (range)	38 (20–80)	57 (34–63)	25 (20–54)	45 (19–85)	50 (41–59)	38 (19–85)	38 (20–82)
Pharmacological immunosuppression	0	0	0	1 (17)	1 (50)	2 (5)	3 (5)
HIV positive	0	0	0	0	0	0	2 (4)
Cancer	3 (14)	3 (60)				6 (15)	1 (2)
Duration of hospital stay, median (range)	12 (5–47)	15 (9–22)	18 (5–97)	10.5 (8–23)	6.5 (6–7)	12 (5–97)	12 (6–79)
Symptoms or clinical signs							
Fever ≥ 38 ^o^C	12 (55)	4 (80)	4 (67)	6 (100)	0	26 (63)^b^	20 (36)
Headache	11 (50)	3 (60)	4 (67)	5 (83)	1 (50)	22 (54)	27 (49)
Altered mental status	20 (91)	4 (80)	4 (80)	6 (100)	2 (100)	36 (88)	47 (85)
Focal neurologic signs	5 (23)	1 (20)	0	3 (50)	0	9 (22)	11 (20)
Seizures	5 (23)	3 (60)	4 (67)			12 (29)	17 (31)
Stiff neck	5 (23)	0	0	2 (33)	0	7 (17)	12 (22)
CSF analysis							
WBC count, cells/mm^2^, median (range)	26 (1–1225)	34 (3–203)	73 (1–792)	79 (1–182)	48 (4–91)	41 (1–1225)^b^	16 (1–362)
Protein level g/L, median (range)	0.54 (0.16–3.21)	0.52 (0.23–0.86)	0.42 (0.27–1.31)	1.00 (0.45–1.73)	0.50 (0.27–0.73)	0.57 (0.16–3.21)	0.58 (0.11–3.33)
Death	0	0	0	1 (17)	0	1 (2.4)	0

^a^Data are provided as number of patients (%) unless indicated otherwise.

^b^p < 0.05 when comparing all patients with identified cause to all patients in whom the cause was not identified; when HHV-1 infected patients were compared to patients with unknown etiology, only the gender remained significantly different; continuous data were compared with Mann–Whitney *U* test, while proportions were analyzed by Fisher's exact test.

There were not enough patients with different etiologies for a meaningful statistical comparison of clinical features and CSF data, but when all patients with identified cause were compared to those with unknown cause the latter group was found to contain more women, was less likely to manifest fever and had lower CSF pleocytosis (p < 0.05). When HHV-1 infected patients were compared to those with unknown etiology, the two groups differed only by gender (Table 2). HHV-1 viral load did not correlate with the time that elapsed between the first symptoms and CSF collection (p = 0.46 by Spearman rank correlation test) nor did it correlate with sample storage duration (p = 0.64 by Spearman rank correlation test). Furthermore, there was no significant difference in time from the onset of first symptoms to CSF collection between patients with identified cause of encephalitis and patients with unknown etiology (median 12 vs 14 days, p = 0.79 by Mann-Whitney U test)

## Discussion

The most frequent cause of encephalitis among our patients was HHV-1, thus confirming the results of a number of previous studies [3,7,8]. Similarly, high prevalence of VZV, another member of herpesviridae family, is also in line with previously published results [3,4,7,8].

High prevalence of Enterovirus infection, second only to HHV-1, was somewhat unexpected. While this pathogen dominates among the causes of meningitis, it is also responsible for encephalitis, but mostly in children. The prevalence of 7.3% found among our patients, all adults, is much higher than that reported in France [8] and England [3], but still lower than that found in the California Encephalitis Project [4]. However, the latter contained a significant proportion (45%) of children. Enterovirus encephalitis is usually mild with the exception of Enterovirus 71 infection, which can cause severe disease [25].

TBEV infection was detected in 6 (6.3%) of patients, which is not surprising, as it has become a major health problem in Europe and Asia and is a common cause of viral brain infection in many countries [26,27]. Over the last few decades the number of reported TBEV encephalitis cases has increased due to a variety of factors including global warming, which extended the length of tick feeding season and its habitat range, as well as to extensive reforestation efforts and to the increase in outdoor activities [27,28]. In Poland, the number of reported cases increased over two-fold [29] and similar upward trends were observed in many European countries [26].

WNV was not detected in any of our patients despite reports of WNV infection in several European countries including Poland's direct neighbors [30]. These findings are in agreement with results of a large study searching for viral sequences in brain tissue from over 2000 wild birds, and suggest that WNV did not get a foothold in Poland as yet [31]. However, the situation could change fast as the mosquito vectors capable of transmitting WNV infection are locally present and the infection is already well established in Europe.

The above results are unlikely to be influenced by our selection of the panel of viral pathogens tested, as it was similar to that used in other large studies with the exception of endemic infections like Toscana virus in the French and Spanish studies [7,8] or St. Louis encephalitis in the California study [4]. Furthermore some, but not all, of these studies searched for Influenza A, measles, JC virus, LCMV and EBV, but the number of identified infections was negligible or even null [3,4,7,8].

While the most frequent causes of encephalitis were similar to those reported in other studies, the mortality was very low as only one death was recorded among our patients (mortality of 1%). This patient was a liver transplant recipient, who died due to severe TBEV encephalitis. In comparison, the mortality was 10% in the French study [8] 12% in the English analysis [3] and 11% in the California Encephalitis Project study [4]. Risk of fatal outcome was previously associated with immunosuppression and concomitant cancer as well as older age [8]. The reasons for such a low mortality among our patients are unclear and could not be explained by a difference in the proportion of immunosuppressed patients: among our patients 7.3% were either HIV-positive or received immunosuppressive drugs and further 7.3% had cancer, while in the English report 15% were immunocompromised [3], in the French study 6.3% were immunosuppressed and 5.5% had cancer [8] and the California study specifically excluded immunocompromised patients altogether [4]. Older age, which was previously associated with poor prognosis [8] was also unlikely to play a role as our patients were older as a group than patients in the above studies. However, an important role could have been played by the absence of bacterial infections among our patients. In the French study [8] both mycobacterium tuberculosis and listeriosis were prominent and characterized by high fatality rates and high mortality was also associated with mycobacterial infection in the English study [3]. While secondary tuberculosis is not uncommon in Poland, all patients with this infection had meningitis only and therefore did not meet the criteria for inclusion into the study. However, it should be mentioned that there were no deaths among our 22 patients with HHV-1 encephalitis. Whether this low mortality is the effect of early and rigorous commencement of Acyclovir therapy or is just an aberration related to small number of cases is unclear.

In 57.3% of our patients the etiology was unclear as neither pathogen sequences nor specific antibodies could be found. This proportion is similar to that reported in other studies where it ranged from 40% to 80% [5,15] although in a recent recent English study it was as low as 37% [3]. This group is likely to be polymorphic as encephalitis could be caused by a plethora of pathogens many of which are rare and therefore rarely tested for or by as yet unknown agents. In addition, viral pathogens are typically present in CSF only transiently and this positivity window could be easily missed, particularly when the spinal tap is done too late in the course of illness [32]. Furthermore, some encephalitis cases could be immune-mediated, such as the acute disseminated encephalomyelitis (ADEM) [33] and encephalitis associated with voltage-gated potassium channel antibodies [34] and NMDA receptor antibodies [35]. While ADEM in our patients was excluded by MRI scans, NMDA receptor and voltage-gated potassium antibodies were not tested. Patients with antibody-associated encephalitis were reported to have absence of fever and only mild CSF pleocytosis and these characteristics were shared by our patients. However, these patients also have poor outcome and prominent seizures [3], which was not the case in our patients. Despite absence of immunomodulatory treatment the clinical outcome was good and no deaths were recorded while in the extensive English study the mortality in the antibody-associated hepatitis was as high as 56% [3]. This absence of seizures and good outcome among our patients suggest a different etiology. Interestingly, while among patients with identified cause the majority were men, in the unknown cause group the majority were women. Further research is needed to elucidate causality in these patients.

In summary, we identified HHV-1 followed by Enterovirus, VZV and TBEV as the most common causes of encephalitis among adult patients in Poland. Importantly, WNV infection was not detected among our patients. In a large proportion of patients the cause of encephalitis was unknown.

## References

[1] Solomon T, Hart IJ, Beeching NJ (2007) Viral encephalitis: a clinician's guide. Pract Neurol 7: 288–305. doi: 10.1136/jnnp.2007.129098 17885268 10.1136/jnnp.2007.129098

[2] Vora NM, Holman RC, Mehal JM, Steiner CA, Blanton J, Sejvar J (2014) Burden of encephalitis-associated hospitalizations in the United States, 1998–2010. Neurology 82: 443–451. doi: 10.1212/WNL.0000000000000086 24384647 10.1212/WNL.0000000000000086

[3] Granerod J, Ambrose HE, Davies NW, Clewley JP, Walsh AL, Morgan D, et al (2010) Causes of encephalitis and differences in their clinical presentations in England: a multicentre, population-based prospective study. Lancet Infect Dis 10: 835–844. doi: 10.1016/S1473-3099(10)70222-X 20952256 10.1016/S1473-3099(10)70222-X

[4] Glaser CA, Honarmand S, Anderson LJ, Schnurr DP, Forghani B, Cossen CK, et al (2006) Beyond viruses: clinical profiles and etiologies associated with encephalitis. Clin Infect Dis 43: 1565–1577. doi: 10.1086/509330 17109290 10.1086/509330

[5] Granerod J, Crowcroft NS (2007) The epidemiology of acute encephalitis. Neuropsychol Rehabil 17: 406–428. doi: 10.1080/09602010600989620 17676528 10.1080/09602010600989620

[6] Graus F, Titulaer MJ, Balu R, Benseler S, Bien CG, Cellucci T, et al (2016) A clinical approach to diagnosis of autoimmune encephalitis. Lancet Neurol 15: 391–404. doi: 10.1016/S1474-4422(15)00401-9 26906964 10.1016/S1474-4422(15)00401-9 PMC5066574

[7] de Ory F, Avellon A, Echevarria JE, Sanchez-Seco MP, Trallero G, Cabrerizo M, et al (2013) Viral infections of the central nervous system in Spain: a prospective study. J Med Virol 85: 554–562. doi: 10.1002/jmv.23470 23239485 10.1002/jmv.23470

[8] Mailles A, Stahl JP, Steering C, Investigators G (2009) Infectious encephalitis in france in 2007: a national prospective study. Clin Infect Dis 49: 1838–1847. doi: 10.1086/648419 19929384 10.1086/648419

[9] Donoso Mantke O, Vaheri A, Ambrose H, Koopmans M, de Ory F, Zeller H, et al (2008) Analysis of the surveillance situation for viral encephalitis and meningitis in Europe. Euro Surveill 13. 10.2807/ese.13.03.08017-en 18445392

[10] Leyssen P, De Clercq E, Neyts J (2000) Perspectives for the treatment of infections with Flaviviridae. Clin Microbiol Rev 13: 67–82, table of contents. 10627492 10.1128/cmr.13.1.67-82.2000 PMC88934

[11] Petersen LR, Carson PJ, Biggerstaff BJ, Custer B, Borchardt SM, Busch MP (2013) Estimated cumulative incidence of West Nile virus infection in US adults, 1999–2010. Epidemiology and Infection 141: 591–595. doi: 10.1017/S0950268812001070 22640592 10.1017/S0950268812001070 PMC9151873

[12] Campbell GL, Hills SL, Fischer M, Jacobson JA, Hoke CH, Hombach JM, et al (2011) Estimated global incidence of Japanese encephalitis: a systematic review. Bull World Health Organ 89: 766–774, 774A-774E. doi: 10.2471/BLT.10.085233 22084515 10.2471/BLT.10.085233 PMC3209971

[13] Griffin DE (2010) Emergence and re-emergence of viral diseases of the central nervous system. Prog Neurobiol 91: 95–101. doi: 10.1016/j.pneurobio.2009.12.003 20004230 10.1016/j.pneurobio.2009.12.003 PMC2860042

[14] Yip CC, Lau SK, Woo PC, Yuen KY (2013) Human enterovirus 71 epidemics: what's next? Emerg Health Threats J 6: 19780 doi: 10.3402/ehtj.v6i0.19780 24119538 10.3402/ehtj.v6i0.19780 PMC3772321

[15] Granerod J, Tam CC, Crowcroft NS, Davies NW, Borchert M, Thomas SL (2010) Challenge of the unknown. A systematic review of acute encephalitis in non-outbreak situations. Neurology 75: 924–932. doi: 10.1212/WNL.0b013e3181f11d65 20820004 10.1212/WNL.0b013e3181f11d65

[16] Infectious diseases and poisonoings in Poland in 2015. National Institute of Public Health-National Institute of Hygiene- Department of Epidemiology

[17] Machura P, Gorka E, Mlynarczyk-Bonikowska B, Majewska A, Malejczyk M, Mlynarczyk G, et al (2015) [Novel multiplex real-time PCR assay for detection and differentiation of herpes simplex virus type 1 and 2 DNA]. Med Dosw Mikrobiol 67: 125–132. 26591664

[18] Dzieciatkowski T, Przybylski M, Gierynska M, Luczak M (2008) [Real-time PCR as an efficient tool for investigating the presence of human herpesvirus 6 DNA]. Med Dosw Mikrobiol 60: 259–265. 19143180

[19] Kierat S, Les K, Przybylski M, Dzieciatkowski T, Mlynarczyk G (2012) [TaqMan fluorescent probe-based real-time PCR assay for detection of varicella-zoster virus]. Med Dosw Mikrobiol 64: 139–149. 23072059

[20] Les K, Przybylski M, Dzieciatkowski T, Mlynarczyk G (2010) [Detection of human enteroviruses with real-time PCR assay using TaqMan fluorescent probe]. Med Dosw Mikrobiol 62: 245–253. 21114017

[21] Rynans S, Dzieciatkowski T, Przybylski M, Basak GW, Rusicka P, Tomaszewska A, et al (2015) Incidence of adenoviral DNAemia in Polish adults undergoing allogeneic haematopoietic stem cell transplantation. Arch Immunol Ther Exp (Warsz) 63: 79–84. 25376263 10.1007/s00005-014-0320-z

[22] Burns MJ, Valdivia H (2008) Modeling the limit of detection in real-time quantitative PCR. Eur Food Res Technol 226: 1513–1524.

[23] Lipowski D, Popiel M, Perlejewski K, Nakamura S, Bukowska-Osko I, Rzadkiewicz E, et al (2017) A Cluster of Fatal Tick-borne Encephalitis Virus Infection in Organ Transplant Setting. J Infect Dis. (in press) 10.1093/infdis/jix040 28453842

[24] Jablonska J, Popiel M, Bukowska-Osko I, Perlejewski K, Caraballo Cortes K, Horban A, et al (2017) No evidence of West Nile virus infection among Polish patients with encephalitis. Centr Eur J Immunol 41: 383–385. 10.5114/ceji.2016.65137 PMC5382883 28450801

[25] Huang CC, Liu CC, Chang YC, Chen CY, Wang ST, Yeh TF (1999) Neurologic complications in children with enterovirus 71 infection. N Engl J Med 341: 936–942. doi: 10.1056/NEJM199909233411302 10498488 10.1056/NEJM199909233411302

[26] Suss J (2011) Tick-borne encephalitis 2010: epidemiology, risk areas, and virus strains in Europe and Asia-an overview. Ticks Tick Borne Dis 2: 2–15. doi: 10.1016/j.ttbdis.2010.10.007 21771531 10.1016/j.ttbdis.2010.10.007

[27] Kunze U (2016) The International Scientific Working Group on Tick-Borne Encephalitis (ISW TBE): Review of 17 years of activity and commitment. Ticks Tick Borne Dis 7: 399–404. doi: 10.1016/j.ttbdis.2015.12.018 26795231 10.1016/j.ttbdis.2015.12.018

[28] Dobler G, Gniel D, Petermann R, Pfeffer M (2012) Epidemiology and distribution of tick-borne encephalitis. Wien Med Wochenschr 162: 230–238. doi: 10.1007/s10354-012-0100-5 22699708 10.1007/s10354-012-0100-5

[29] Polkowska A (2011) [Meningitis and encephalitis in Poland in 2009]. Przegl Epidemiol 65: 213–218. 21913462

[30] Chancey C, Grinev A, Volkova E, Rios M (2015) The global ecology and epidemiology of West Nile virus. Biomed Res Int 2015: 376230 doi: 10.1155/2015/376230 25866777 10.1155/2015/376230 PMC4383390

[31] Niczyporuk JS, Samorek-Salamonowicz E, Mizak WK (2011) The survey of wild birds for West Nile virus in Poland. Pol J Vet Sci 14: 573–577. 22439327 10.2478/v10181-011-0085-9

[32] Davies NW, Brown LJ, Gonde J, Irish D, Robinson RO, Swan AV, et al (2005) Factors influencing PCR detection of viruses in cerebrospinal fluid of patients with suspected CNS infections. J Neurol Neurosurg Psychiatry 76: 82–87. doi: 10.1136/jnnp.2004.045336 15608000 10.1136/jnnp.2004.045336 PMC1739313

[33] Sonneville R, Klein I, de Broucker T, Wolff M (2009) Post-infectious encephalitis in adults: diagnosis and management. J Infect 58: 321–328. doi: 10.1016/j.jinf.2009.02.011 19368974 10.1016/j.jinf.2009.02.011 PMC7125543

[34] Vincent A, Buckley C, Schott JM, Baker I, Dewar BK, Detert N, et al (2004) Potassium channel antibody-associated encephalopathy: a potentially immunotherapy-responsive form of limbic encephalitis. Brain 127: 701–712. doi: 10.1093/brain/awh077 14960497 10.1093/brain/awh077

[35] Dalmau J, Gleichman AJ, Hughes EG, Rossi JE, Peng X, Lai M, et al (2008) Anti-NMDA-receptor encephalitis: case series and analysis of the effects of antibodies. Lancet Neurol 7: 1091–1098. doi: 10.1016/S1474-4422(08)70224-2 18851928 10.1016/S1474-4422(08)70224-2 PMC2607118

